# Myelin Pathology in Alzheimer's Disease: Potential Therapeutic Opportunities

**DOI:** 10.14336/AD.2023.0628

**Published:** 2024-04-01

**Authors:** Zhihai Huang, J. Dedrick Jordan, Quanguang Zhang

**Affiliations:** Department of Neurology, Louisiana State University Health Sciences Center, Shreveport, LA 71103 USA.

**Keywords:** Alzheimer's disease (AD), myelin, oligodendrocyte, cognition

## Abstract

Alzheimer's disease (AD) is an age-related neurodegenerative disease characterized by memory loss and cognitive decline. Despite significant efforts over several decades, our understanding of the pathophysiology of this disease is still incomplete. Myelin is a multi-layered membrane structure ensheathing neuronal axons, which is essential for the fast and effective propagation of action potentials along the axons. Recent studies highlight the critical involvement of myelin in memory consolidation and reveal its vulnerability in various pathological conditions. Notably, apart from the classic amyloid hypothesis, myelin degeneration has been proposed as another critical pathophysiological feature of AD, which could occur prior to the development of amyloid pathology. Here, we review recent works supporting the critical role of myelin in cognition and myelin pathology during AD progression, with a focus on the mechanisms underlying myelin degeneration in AD. We also discuss the complex intersections between myelin pathology and typical AD pathophysiology, as well as the therapeutic potential of pro-myelinating approaches for this disease. Overall, these findings implicate myelin degeneration as a critical contributor to AD-related cognitive deficits and support targeting myelin repair as a promising therapeutic strategy for AD.

## INTRODUCTION

Alzheimer's disease (AD) is a major public health challenge, affecting millions of people worldwide. With the population aging, the prevalence of AD has risen sharply in recent years [1]. Unfortunately, there is currently no effective intervention that prevents or halts the progression of AD, and its pathogenesis remains incompletely understood. Historically, extracellular amyloid plaques and neurofibrillary tangles, two hallmark features of AD, were suggested to be involved in the pathogenesis of this disease [2]. However, most therapeutic interventions targeting amyloid beta (Aβ) removal have resulted in disappointing clinical results. This has led to a paradigm shift in AD research, and several other mechanisms have been proposed to explain the pathophysiology of AD [3, 4]. This is especially true with recent studies highlighting the potential contribution of myelin degeneration in the progression of AD.

In the central nervous system (CNS), myelin is a multilayer membrane isolating the neuronal axons from the surrounding environment, which allows the effective propagation of action potentials along the axons [5]. Furthermore, well-functioning myelin metabolically supports axon integrity and several critical neurological functions. Multiple lines of evidence have shown that myelination, the process involving the generation of new myelin, is required for memory consolidation and cognition [6, 7]. Furthermore, myelin degeneration has been reported in a variety of neurodegenerative disorders, including cognitive impairment and AD. Widespread myelin impairment has been observed in pre-clinical AD patients, a pathology that may precede classic amyloid pathology [8]. Conversely, enhanced myelination has been shown to rescue aging and AD-related cognitive deficits in rodent models, suggesting the critical role of myelin in maintaining cognition [9].

This review summarizes the physiological action of myelin in the CNS and the current understanding of myelin pathologic alterations during AD progression. Numerous studies now indicate that myelin degeneration is involved in the pathogenesis of AD and is related to cognitive impairment. Additionally, pro-myelinating approaches have shown encouraging results in preclinical studies. Based on these findings, we propose that myelin repair could be a promising therapeutic target for AD.

## The crucial role of myelin in memory consolidation and cognition

**Figure 1.**
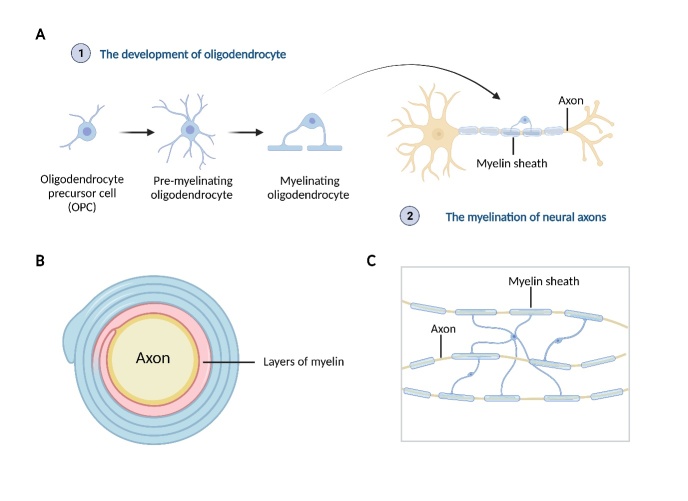
**Schematic depiction of oligodendrocyte and myelination**. **(A)** The developmental process of oligodendrocytes and the myelination of neural axons. **(B)** A cross-section view of myelinated axon, where multiple myelin segments from various myelinating oligodendrocytes constitute the layers of myelin (C) Numerous myelin sheaths are produced by oligodendrocytes in the CNS to enclose various neural axons.

Oligodendrocytes are specialized myelin-forming cells in the CNS, which represent the second major glial cell type in the mammalian CNS [10]. Although most oligodendrocytes were formed early in life, the renewal of oligodendrocyte/myelin occurs throughout life and declines with aging. Myelination is a complicated physiological process that is regulated by various molecular signals. First, oligodendrocyte precursor cells (OPCs) proliferate and migrate to selective brain regions, where they undergo differentiation to become immature oligodendrocytes [11]. Immature oligodendrocytes then undergo a series of maturation steps to develop into mature and functioning oligodendrocytes [5, 11]. Typically, mature myelin wraps around the axons to act as an insulator to ensure rapid and effective nerve conduction (Fig. 1) [12, 13]. Each oligodendrocyte in the CNS system produces multiple sheaths to surround neuronal axons, in contrast to Schwann cells in the peripheral nervous system that create a single myelin sheath around the axon [5]. Myelinated axons also lead to more efficient nerve impulse transmission while the pathologic disruption of axonal myelin significantly increases the energy requirements of neurons to maintain normal neurotransmission levels [14, 15]. Moreover, the myelin sheath exerts significant influences on both neuronal survival and synaptic function. Studies have shown that exposure to OPCs or oligodendrocyte-conditioned media can increase the survival of neuronal cultures, an effect mediated by the PI3kinase/Akt pathways [16]. Conversely, deficits in myelin have been found to disrupt neuronal excitability and lead to aberrant neuronal communication [17, 18].

In addition, by modulating neurotransmission and synaptic plasticity, myelin is implicated in the regulation of synaptic function [19, 20]. Myelin dysfunction resulting from chronic hypoxia has been shown to cause profound synaptic deficits, while enhanced myelin renewal effectively reversed these deficits [21]. Aberrant neural communication and synaptic functions have also been observed in the cuprizone-induced demyelination model. Local injection of cuprizone or diet cuprizone feed resulted in massive brain demyelination, coinciding with the loss of neurons, disrupted neuronal communication, and synaptic deficits [22-24]. Collectively, these findings highlight the pivotal role of the myelin sheath in coordinating neuronal survival, communication, and synaptic functions.

**Figure 2.**
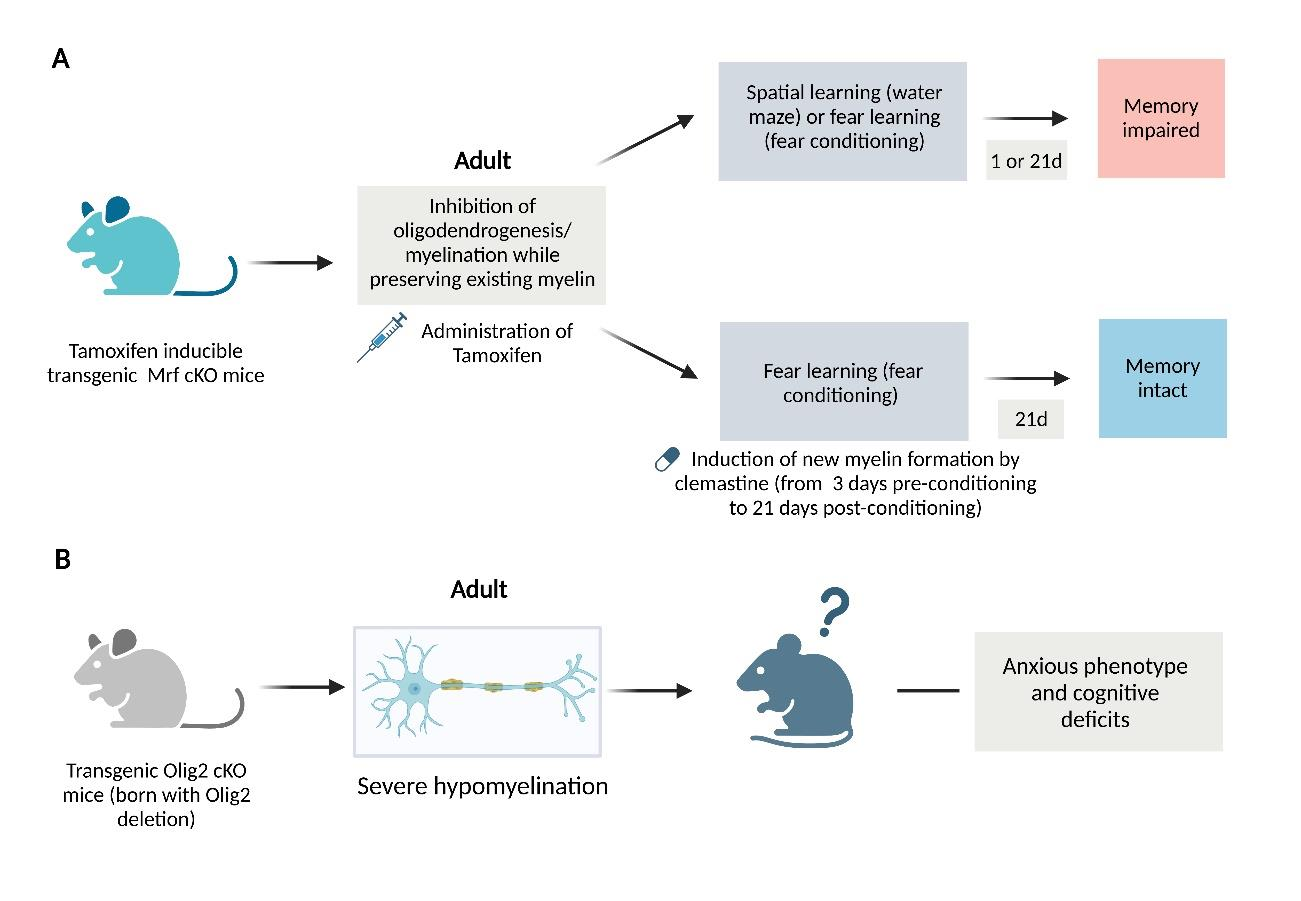
**The critical role of myelin in memory and cognition**. **(A)** The tamoxifen-inducible transgenic Mrf cKO mice exhibit inhibited oligodendrogenesis/ myelination after spatial learning or fear learning. These animals also exhibit impaired memory. Additionally, the induction of new myelin formation rescues the compromised long-term fear memory resulting from Mrf ablation. **(B)** The mice born with Olig2 deletion show severe hypomyelination, anxious phenotype, and cognitive deficits in adulthood.

Recent works in experimental animals have highlighted the pivotal role of myelin in memory consolidation and cognitive function. Steadman and colleagues reported that the learning task promotes oligodendrogenesis (the process of generating new oligodendrocytes) and myelination in mice [6]. Further studies demonstrated that the inhibition of new myelin formation by deleting the Mrf gene (also known as Myrf, a critical transcription factor required for oligodendrocytes to initiate and maintain the myelination program), disrupted learning task-motivated oligodendrogenesis and impeded the consolidation of spatial learning [6]. Additionally, the formation of new myelin is crucial for the preservation of remote fear memory [20]. The mice lacking Mrf in OPCs failed to generate new myelin after the fear conditioning task and were unable to retain the fear memory over a longer period of time, whereas mice with intact myelin formation showed preserved long-term fear memory [20]. Furthermore, enhancing new myelin formation using a pharmacological approach rescued the deficit in long-term fear memory [20]. These findings suggest oligodendrogenesis/myelination is essential for memory consolidation. Along the same lines, the ablation of oligodendrocyte transcription factor 2 (Olig2), a transcription factor controlling oligodendrocyte development, resulted in hypomyelination and cognitive deficits in mice [7, 25]. Intriguingly, the Olig2 cKO mice also exhibited anxiety-like behaviors, a common comorbidity of AD, at the young adult stage of life [7, 26]. The key findings of these works are depicted in Figure 2. The observation that myelin degeneration is seen in a wide variety of neurodegenerative diseases, has prompted us to reevaluate the contribution of oligodendrocyte/myelin pathology in the pathogenesis of these diseases.

## Myelin degeneration as an early pathological event in the progression of AD

Oligodendrocytes and myelin are vulnerable to various pathological conditions, including hypoxia, ischemia and brain insults [27-29]. Furthermore, compromised oligodendrocyte and myelin degeneration have been observed in patients with multiple sclerosis, Parkinson's disease, Huntington's disease, and amyotrophic lateral sclerosis [30-33]. Similarly, myelin degeneration has been extensively reported in both experimental animal models of AD and patients with AD. Observations related to myelin pathology date back several decades, and have indicated that myelin, and several lipids essential for maintaining myelin integrity, are significantly reduced in the brain tissue of patients with AD [34, 35]. These findings were further supported by another study showing that there was a remarkable decrease in the amount of myelin basic protein (MBP), myelin proteolipid protein in the postmortem brains of patients with AD [36]. Notably, cholesterol, an essential component of myelin sheaths, was also found to be diminished, accompanied by elevated levels of Aβ peptides [36]. Another study conducted by Zhan and colleagues investigated myelin loss and degradation in AD [37]. The results showed that in comparison to the brain of age-matched healthy individuals, the brains of patients with AD exhibited significant loss of myelin integrity and higher levels of myelin degradation [37].

Recent advancements in sophisticated brain-imaging techniques have enabled us to better assess myelin changes in AD. Analysis of the T2-weighted magnetic resonance imaging (MRI) has revealed a marked reduction in myelin content in the parietal and temporal cortices of patients with AD and mild cognitive impairment compared to controls [38]. Furthermore, this reduction in myelin content was more pronounced in patients with AD than in those with mild cognitive impairment [38]. The study also found that the decrease in myelin content was correlated with increased age and greater neuropsychiatric impairment severity [38]. The recent development of q-Space Myelin Map Imaging, an MRI technique, holds promise for further characterization of myelination. This approach utilizes a special type of MRI sequence to measure the diffusion of water molecules within myelin, providing a more sensitive way to evaluate the distribution and density of myelin across different brain regions [39]. A neuroimaging study utilizing q-Space Myelin Map Imaging revealed significant alterations in myelin organization in patients with AD [40]. Specifically, AD patients showed decreased myelin density in several brain regions, including the hippocampus, insula, precuneus, and anterior cingulate regions [40]. Furthermore, these alterations in myelin organization were found to correlate with cognitive impairment in these patients with AD. Additional clinical investigations further support these observations [8, 41]. Consistent with these findings, a recent study also revealed reduced MBP expression in the cortex and hippocampus, two brain regions vital for memory and cognition, in brain tissues of patients with AD [42].

Several preclinical animal studies also provide novel insights into the myelin pathology associated with AD [43-45]. The 3xTg-AD mice exhibited remarkable alterations in myelination patterns and oligodendrocyte process in the hippocampus, including diminished mature oligodendrocytes and compromised overall myelinated area [43]. Similarly, early myelin degeneration (at 3-6 months of age) has been reported in the APP/PS1 mice, another transgenic mouse model of AD, and this pathology was observed prior to the significant cognitive deficit (at 6 months of age) [44]. Of note, using R1.40 mice, an APP transgene-based rodent model of AD, Tse et al. found widespread loss and degeneration of oligodendrocytes in the neocortex, accompanied by down-regulated Myrf gene [8]. Further investigations support that myelin loss (at 12 months of age of ages in AD mice) occurs prior to amyloid pathology (at 13-14 months of age in AD mice), suggesting its potential involvement in the pathophysiology of AD [8]. Despite these promising findings however, the clinical data regarding the temporal relationship between myelin degeneration and amyloid deposition/tau pathology is still lacking. Human brain samples offer insights into specific stages of the disease; however, human brain sample only provide a snapshot of a specific stage of the disease and is affected by high variability. Furthermore, the dynamic nature of AD progression complicates the interpretation of these findings. Therefore, the identification of the temporal sequence of these cellular events in AD patients remains challenging. Notably, through the analysis of postmortem brain samples from patients across various age stages, a report has highlighted a defect in myelin lipid biosynthesis during the preclinical stages of the disease, which preceded the appearance of tau pathology in the cortical regions typically affected by the disease [46].

Intriguingly, although the AD brain typically exhibits extensive oligodendrocyte/myelin dysfunction, the OPCs exhibit a high proliferative rate in the context of AD [42]. A recent study that utilized transgenic mouse models, has enabled the visualization of pre-existing myelin and newly formed myelin, providing new insights into the myelin maintenance and repair mechanisms during AD progression [42]. In comparison to age-matched wildtype animals, the AD mice exhibited extensive demyelination and diminished stable myelin, while displaying increased new myelin. The increase in new myelin may represent an endogenous repair mechanism in response to extensive demyelination [42, 47]. Nevertheless, this compensatory proliferation and repair may remain limited and therefore fail to restore compromised neural networks affected by myelin destruction. Furthermore, amyloid and tau deposition show overlap with the myelin repair pathway [48, 49]. A variety of signaling pathways involved in myelin repair are also compromised in the early stages of AD, as discussed in a previous review [49]. These findings highlight the potential connection between myelin dysfunction and AD pathophysiology. Moreover, inhibiting myelin renewal can lead to spatial memory deficits in young animals, resembling those observed in aging, whereas enhancing new myelin generation has been shown to prevent memory deficit during aging [25]. In addition, increased levels of tau protein in the cerebrospinal fluid have been observed in both experimental models of multiple sclerosis and patients with multiple sclerosis, an autoimmune-mediated demyelinating disease [50-52]. It is noteworthy that patients with multiple sclerosis often exhibit synapse loss and cognitive deficits [53, 54]. The deficiency in myelin sulfatide, a critical component of the myelin sheath essential for its structure, function, and integrity, can lead to AD-like neuroinflammation and cognitive impairment [55]. A more recent study demonstrated that when mice carrying a mutation associated with myelin defects were crossed with transgenic mice, the offspring exhibited early-onset deposition of Aβ in the brain, in comparison to age-matched controls [56]. Furthermore, it has been observed that in elderly individuals with a positive Aβ PET scan, higher levels of myelin are associated with a lower susceptibility to accumulate fibrillar tau [57]. These findings emphasize the potential contribution of myelin dysfunction in AD pathophysiology. The exact role of myelin pathology in AD progression, as well as its potential interactions with other factors associated with AD, still requires further investigation.

Collectively, these observations highlight myelin degeneration as an early event and significant pathological feature in AD progression, which may contribute to disease-related cognitive decline.

## Molecular basis underlying myelin degeneration during AD progression

In the CNS, oligodendrocytes are highly susceptible to insults for several reasons. First, oligodendrocytes are responsible for the production and maintenance of the myelin sheath, which represents an energetically demanding process [58]. As a result, the energy requirements of oligodendrocytes are 2 to 3-fold higher than other brain cells [59]. In this context, when oligodendrocytes are exposed to injury, they are unable to meet the increased metabolic demands, leading to cellular dysfunction and death. Second, oligodendrocyte linage cells, especially OPCs, contain a low level of antioxidant defense compared to other glial cells. Indeed, oligodendrocyte linage cells only express low amounts of the antioxidative enzyme glutathione, which makes them particularly vulnerable to oxidative damage [60, 61]. Third, oligodendrocytes are the cells that contain the highest iron levels in the brain [59]. Although proper iron metabolism is required for the physiological process in oligodendrocytes, aberrant iron homeostasis under pathological situations may exacerbate oxidative stress in oligodendrocyte linage cells and lead to cell damage [59, 62].

**Figure 3.**
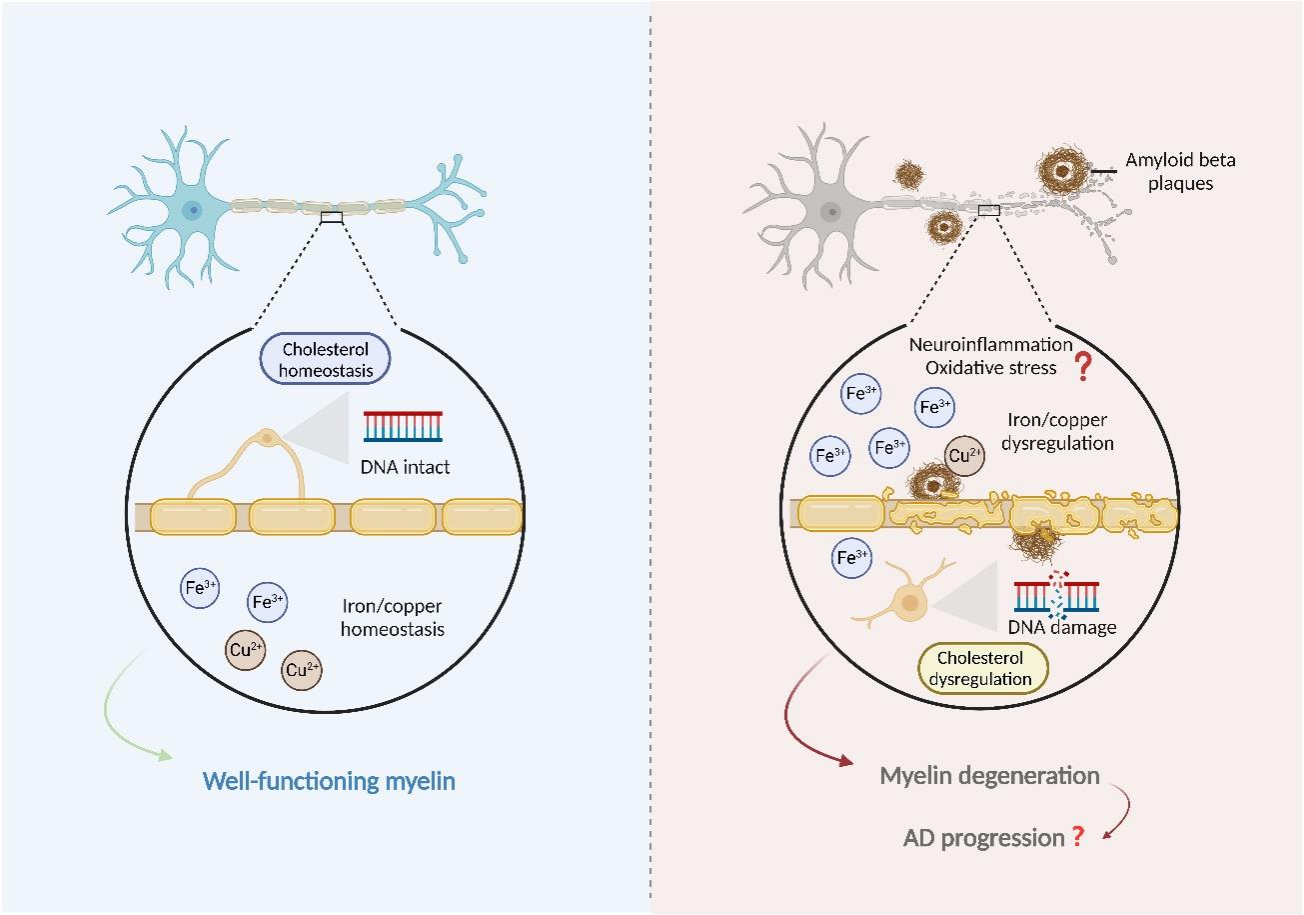
**The potential mechanisms underlying myelin degeneration during AD progression**. Under normal physiological conditions (left), the brain maintains cholesterol and iron/copper homeostasis to support the integrity of myelin and the proper myelination process. In the context of AD (right), oligodendrocyte lineage cells exhibit DNA damage, which prevents them from developing into myelinating oligodendrocytes. Additionally, the toxic effects of amyloid beta, as well as cholesterol dysregulation, and iron/copper dyshomeostasis, may also contribute to myelin breakdown and subsequent axonal degeneration. Furthermore, other mechanisms including neuroinflammation and oxidative stress may also involve in myelin dysfunction.

Since AD is associated with oxidative stress and metal iron overload [63, 64], it is tempting to hypothesize that oligodendrocyte/myelin may be the primary target for these pathologic events. Indeed, recent studies have provided insights into the etiology of oligodendrocyte/myelin degeneration during AD progression (Fig. 3). We discuss these research advances in detail in the following sections.

### 1) DNA damage and Aβ toxicity

A growing body of research has suggested that DNA damage is implicated in aging and neurodegenerative diseases including AD [65, 66]. Several observations have also proposed that DNA damage might be among the earliest detectable events in the progression of AD [67, 68]. However, the pathological link between DNA damage and myelin degeneration has only recently been established. Tse and colleagues first reported that DNA damage in oligodendrocytes was an early event in the development of AD, which occurred before neurodegenerative changes including the accumulation of amyloid plaques in the brain [8]. Further analysis of postmortem brain tissues from patients with AD also revealed significant DNA damage and oligodendrocyte degeneration [8]. To gain more insights into the contribution of DNA damage to oligodendrocyte dysfunction, primary MBP-expressing oligodendrocyte cultures were treated with etoposide to induce DNA damage. The results showed that etoposide remarkably increased the expression of nuclear foci of gH2A.X and 53BP1, markers of DNA double-strand breaks, in the cultures and diminished the number of MBP-expressing oligodendrocytes in a concentration-dependent manner [8]. These findings indicate that DNA damage can directly cause oligodendrocyte dysfunction and prevent the formation of myelinating oligodendrocytes. Given that inflammation and amyloidosis also represent the prominent pathologic feature of AD, further testing was performed to determine the effects of lipopolysaccharide or a soluble mixture of Aβ^25-35^ on the oligodendrocyte cultures. However, neither lipopolysaccharide nor Aβ^25-35^ exposure led to changes in the number of MBP-positive cells in oligodendrocyte cultures, suggesting that inflammation and amyloidosis might not directly contribute to oligodendrocyte dysfunction [8].

Similarly, the neurotoxic effects of Aβ on oligodendrocyte myelin have been wildly reported [43, 69, 70]. A study conducted using purified oligodendroglial cultures at various developmental stages has demonstrated that high concentrations of Aβ oligomers can significantly decrease the survival of mature oligodendrocytes [70]. Notably, a more recent study suggested that the treatment of soluble Aβ^1-42^ does not alter the generation of new myelin in mice and does not affect the MBP-positive cells and OPCs *in vitro* [71]. Notably, Aβ can exist in different forms, including plaque and soluble forms. Different from soluble forms, the accumulation of Aβ plaque may play a role in the demyelination process. In both human and transgenic AD animals, demyelination was detected in many brain areas critical for cognition and was most pronounced at the core of amyloid plaques [71, 72]. In contrast, no significant myelin loss was observed in plaque-free regions in comparison to the controls [72]. Hence, Aβ-mediated toxicity may have a profound effect on oligodendrocytes/myelin, but the relative pathogenic importance of different forms of Aβ in these physiological processes remains incompletely understood.

### 2) Cholesterol dysregulation

There has been an increasing appreciation for the role of cholesterol in myelin formation. Indeed, cholesterol homeostasis in the brain not only influences myelin development but is also essential to maintain proper myelin function [73]. The significance of cholesterol in myelination was indicated in a study employing mice that are incapable of synthesizing cholesterol in myelin-forming oligodendrocytes [74]. Although these mutant cells were still able to survive, the deficient cholesterol in oligodendrocytes led to extensive hypomyelination in the brain [74]. Furthermore, despite the appropriate level of cholesterol is critical for myelin processes, the excessive accumulation of cholesterol can also compromise myelination. Lysolecithin-induced focal demyelination is accompanied by the aggregation of cholesterol in the myelin debris, whereas by enhancing cholesterol efflux and solubility using cyclodextrin, the regeneration of myelin in the lesion area was promoted [75]. Aberrant expression of genes involved in brain cholesterol metabolism has also been observed in AD and other neurodegenerative diseases [76, 77].

Intriguingly, a recent study shed new light on the role of Apolipoprotein E4 (APOE4), a major genetic risk factor associated with AD, in AD-linked cholesterol dysregulation and demyelination [78]. Firstly, they demonstrated that the human brains from APOE4 carriers show increased intracellular accumulation of cholesterol, fewer myelinated axons, and thinner myelin sheaths in comparison to age-matched non-carriers [78]. Further analyses indicated that APOE4 strikingly altered signaling pathways related to cholesterol transport and cellular cholesterol homeostasis. Next, using oligodendrocyte and APOE4-expressing oligodendrocyte cultures, they found that APOE4 in oligodendrocytes contributes to cellular cholesterol accumulation and diminished fluorescence intensity of MBP [78]. Notably, the administration of cyclodextrin, a molecule known to reduce intracellular cholesterol accumulation, reduced cholesterol burden, and increased axonal myelination in APOE4 mice [78]. Correspondingly, these animals also show improved learning and memory performances in cognitive tasks.

Notably, while APOE4-allele could increase the risk of AD, not all cases of AD are linked to the APOE4-allele. Indeed, only 50% of AD cases carry an APOE4 allele, but aberrant cholesterol metabolism has also been observed in the absence of APOE4 [79, 80]. In this regard, further investigations are required to identify other mechanisms underlying the aberrant cholesterol metabolism in AD. A comprehensive understanding of these physiological processes will potentially facilitate the development of disease-modifying strategies.

### 3) Iron/copper dyshomeostasis

Metal ions such as copper and iron represent important signals during various physiological processes in the brain, which also play key roles in the maintenance and function of myelin [62]. As a necessary co-factor for cholesterol and lipid biosynthesis, iron is directly involved in the physiological process of myelination [59]. Copper, on the other hand, is also critical for proper myelin growth and axon-myelin maintenance [81]. Compared to age-matched controls, iron storage protein ferritin knockout mice had decreased proteolipid protein, the major integral membrane protein of CNS myelin, paralleled by fewer total myelin proteins [82]. Similarly, the rats fed with an iron-deficient diet showed decreased brain iron content and altered myelin composition, with a 30% decrease in cholesterol in the brain white matter [82]. Myelin pathology, including demyelination, delayed myelination, and subsequent cavitation of the white matter, has also been found in copper-deficient animals [83, 84]. These deficiencies can be partially reversed by subsequent copper diet supplements [85]. The adverse consequences of copper deficiency are well-recognized in human diseases. Menkes' disease is a copper-deficient disease caused by mutations in genes coding for the copper-transport protein ATP7A. The generalized copper deficiency leads to widespread myelin loss and downregulation of genes coding myelin metabolism [86, 87]. Furthermore, a case report demonstrated CNS demyelination in a patient that received excessive treatment with the copper antagonist penicillamine [88].

Conversely, excessive amounts of iron and copper can also be toxic to the brain and may contribute to neurodegeneration. Transgenic mice with chronic iron loading exhibited diminished myelin-related genes in the corpus callosum, despite the overall myelin structure and integrity remaining unaffected [89]. Likewise, excessive iron intake led to reduced expression of MBP, a marker of mature oligodendrocytes, in the hippocampus of nursing piglets [90]. An MRI study conducted in older adults showed that increased levels of iron in various brain regions were associated with reduced myelin content, which can be a predictor of memory loss during aging [91]. Correspondingly, the toxic effects of overloaded copper on myelin have been increasingly recognized. Zhang et al. investigated how copper stress affects the physiological process of myelin in zebrafish [92]. The copper exposure caused compromised myelin development and significant axon defects in the zebrafish embryos, indicating the role of excessive copper in myelin dysfunction [92]. Therefore, normal myelin development may be tightly regulated by iron/copper homeostasis.

Abnormal levels of copper and iron have been extensively observed in the brains of AD patients. Studies using MRI have indicated that patients with AD show higher concentrations of iron in various injuries to the brain, including areas for cognition and memory, in comparison to healthy individuals [93-95]. Rather than brain neuropathology such as amyloid plaques and neurofibrillary tangles, the increase in brain iron was associated with the rate of cognitive decline [96]. Moreover, desferrioxamine, an FDA-approved iron chelator, has shown the potential to slow the progression of AD [97]. The change of copper levels in patients with AD is more complicated. Like iron, copper can trigger the aggregation of amyloid plaques and has been found to be highly enriched in the amyloid plaques [98, 99]. Copper binding to amyloid plaques also functions as a catalyst in the production of reactive oxygen species [100]. However, copper seems to be detrimental only when combined with amyloid plaques, and a decrease in plasma and brain copper concentration was normally reported in the cases of AD. A negative correlation between plasma copper levels and cognitive function has been reported in clinically confirmed AD patients [101]. Compared to patients with moderate levels of plasma copper, patients with low plasma copper levels exhibited poorer cognitive functioning composite scores [101]. Moreover, a severe copper deficiency was observed in brain samples from individuals with AD, including a 52.8 to 70.2% reduction in copper levels in the cerebellum, hippocampus, and cortex, compared to that from healthy controls [102]. These reductions in copper levels are as severe as those seen in patients with Menkes' disease, a disorder characterized by copper deficiency [103]. Furthermore, a community-based study found that higher composite brain copper levels were linked to slower cognitive decline [102]. Lastly, individuals who consumed copper-rich diets were found to display slower cognitive decline, particularly those in the middle and highest tertiles of dietary copper intake [104].

Overall, these findings raise the possibility that dysregulation of metal ion homeostasis, including iron and copper, may contribute to AD-linked myelin pathology. To date, however, direct experimental evidence for this hypothesis is lacking, which warrants further investigations. Additionally, although excess zinc burden has also been observed in AD [105], the specific role of zinc in disease-linked myelin degeneration, however, remains to be elucidated. Therefore, zinc dysregulation was not discussed in this review.

### 4) Other potential mechanisms

Besides the main mechanisms discussed above, other potential mechanisms may also contribute to myelin degradation in the context of AD. Neuroinflammation and oxidative stress are common pathological events during the progression of AD [106, 107]. The activation of various inflammatory signaling pathways and the accumulation of reactive oxygen species (ROS) are characteristic features of AD progression. These events can synergistically contribute to cellular damage and dysfunction [108]. Increasingly, the significant impact of neuroinflammation and oxidative stress on the physiological processes of oligodendrocytes and myelin has been recognized. While proper inflammation is required for oligodendrocyte regeneration and myelin repair under various physiological conditions [109, 110], the persistent inflammatory response can hinder oligodendrocytic cell processes [111-113]. The inflammatory molecule S100B protein has been shown to impede the differentiation, maturation, and function of oligodendrocytes in both *in vitro* and *in vivo* models [111]. Additionally, oligodendrocyte lineage cells are highly vulnerable to oxidative stress [61, 114]. Oxidative stress impairs mitochondrial function in oligodendrocyte progenitor cells and hinders their differentiation while these effects can be rescued by antioxidant drug treatment [115]. These findings support the potential involvement of inflammation and oxidative stress in the myelin pathology observed in AD.

However, direct experimental evidence supporting the direct contribution of inflammation/oxidative stress directly to AD-associated myelin degradation is currently lacking. Further investigations, therefore, are required to determine whether and how inflammation and oxidative stress may impact myelin pathology during AD progression.

## The complex interactions between myelin pathology and typical AD pathology

**Figure 4.**
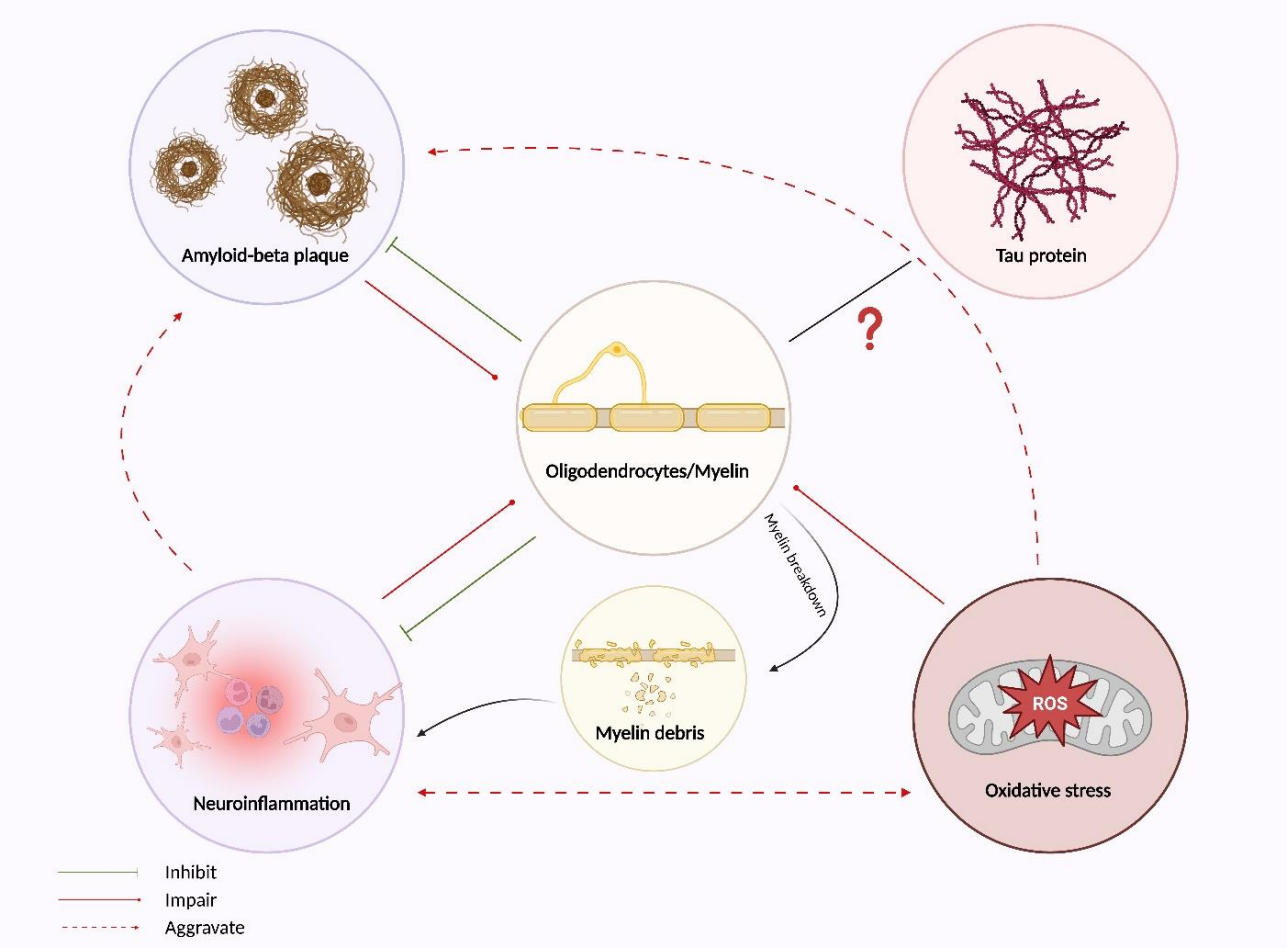
**The complex interactions between myelin pathology and typical AD pathology**. Typical AD pathology includes Aβ, neuroinflammation, and oxidative stress may interfere with myelin sheaths. Myelin degradation as a result of Aβ plaque acculturation, but it can also protect against Aβ toxicity. Additionally, myelin defect could be linked to neuroinflammation, and myelin sheaths may also potentially suppress inflammatory processes. Moreover, the development of oligodendrocytes and myelination could also be hampered by oxidative stress. The interaction of oxidative stress and inflammation may further aggravate Aβ burden and myelin dysfunction. Also, myelin debris resulting from myelin breakdown may trigger pro-inflammatory signaling, leading to a vicious cycle. The potential crosstalk between tau pathology and myelin, however, remains to be determined.

Although recent scientific advancements have contributed to our understanding of the myelin pathology in AD, there remains a significant knowledge gap concerning the intricate connections between myelin pathology and the typical AD pathophysiology (Aβ deposition and tau hyperphosphorylation). Moreover, the potential interplay and dynamic interactions between these cellular events remain poorly understood. As mentioned above, Aβ-mediated neurotoxicity could significantly affect oligodendrocyte functions and myelin integrity [43, 69, 70]. Other AD-associated pathology, including neuroinflammation and oxidative stress, may also impair the development of oligodendrocytes and myelination [111, 115]. Furthermore, neuroinflammation and oxidative stress are inextricably related and can influence each other. Inflammatory processes can promote the generation of ROS, while oxidative stress can also activate proinflammatory signaling pathways [116]. The activation of pro-inflammatory signaling and ROS could further trigger the acculturation of Aβ, which may thus exacerbate myelin defect [117, 118]. Notably, myelin sheaths appear to not only be affected in the pathology of AD, but they may also positively against Aβ pathology and neuroinflammation. Previous reports have shown that MBP, the major structural protein component of myelin, can assemble fibrillar Aβ, and inhibit its cytotoxic effects [119-121]. Furthermore, a recent study provided conclusive evidence for the first time that myelin dysfunction drives Aβ deposition [56]. In 5×FAD mice with mutants that cause myelin abnormalities, exacerbated Aβ deposition was observed at an early age. Intriguingly, acute demyelination induced by a cuprizone-containing diet resulted in similar pathological alternations, suggesting myelin dysfunction may exacerbate the formation of Aβ plaque [56]. Myelin also serves as an inflammatory mediator, myelin lipids and the phagocytosis of myelin contributes to the suppression of macrophage and microglia-mediated inflammatory processes [122, 123]. Myelin debris resulting from myelin breakdown, nevertheless, could trigger or exacerbate the inflammatory response. The intracerebral injection of myelin debris directly induced profound inflammation in the mouse brain [124]. These observations highlight the intricate pathophysiology underlying the role of myelin in the progression of AD progression. These proposed mechanisms and their potential interactions are presented in Figure 4. Additionally, although hyperphosphorylated tau also presents a key hallmark of AD and plays a critical role in AD pathology, the direct relationship between tau pathology and myelin dysfunction, as well as the potential interplay, has not been determined.

In summary, these recent scientific advancements have unveiled the multifaceted role of myelin pathology in the pathophysiology of AD and underscored the imperative for exploring the intricate mechanisms underlying AD from novel perspectives.

## Myelin repair as a potential therapeutic target for AD

The therapeutic potential of pro-myelinating approaches for age-related cognitive decline and AD has garnered growing research interest. Considering the critical role of myelin in cognition and observed myelin degeneration during AD progression, Chen et al. investigated whether enhanced myelin renewal could reverse cognitive deficits in APP/PS1 mice [42]. Firstly, mice with conditional deletion of Chrm1, the gene encoding M1R (a negative regulator of OPC differentiation), in OPCs were crossed with APP/PS1 mice to generate APP/PS1/M1R cKO mice. Compared to age-matched APP/PS1 mice, the APP/PS1/M1R cKO mice exhibited increased myelination in various brain regions, coinciding with better performances in cognitive tasks [42]. Next, they further tested whether clemastine, an anti-muscarinic drug with pro-myelinating properties, could exert similar benefits in aged APP/PS1 mice. In parallel, 3-month clemastine treatment (10 mg/kg/day) significantly boosted the formation of new myelin and these animals also showed improved cognitive performances compared to animals treated with placebo [42]. It is noteworthy that neither the deletion of M1R in OPCs nor the administration of clemastine altered amyloid pathology or microglial physiology in these animals [42]. This suggests that the improvement in cognitive function observed was directly related to myelin repair. Together, for the first time, these findings have directly provided evidence that myelin degeneration serves as a key contributor to AD-linked cognitive deficit and that myelin repair represents a promising therapeutic opportunity for AD.

Clemastine, an FDA-approved therapy for allergies, has also been used in other neurodegenerative disorders. In patients with multiple sclerosis, treatment with clemastine (10.72 mg/day, lasted for 60-90 days) mitigated chronic demyelinating injury, without serious adverse events [125]. The benefits of clemastine have also been reported in a cuprizone-induced demyelination animal model in which clemastine administration promoted remyelination and rescued cognitive impairment associated with myelin loss [126]. Since clemastine is already FDA-approved in humans and has met clinically defined efficacy endpoints in patients, the positive effects of this agent for AD may allow for a prompter translation to treatment. However, before this can occur, a few caveats need to be addressed. For example, the dose of clemastine used in mice was roughly 100 times the maximum recommended dose for humans [9]. Therefore, for a better and safer clinical translation, further studies are warranted to determine the optimal dosage of this agent for patients with AD. Several other pharmacological interventions have also demonstrated the potential in promoting myelination. One such medication is the recently identified myelin-enhancing drug (±)U-50488 [127]. This drug has been proven to promote myelin renewal in experimental animal models of chronic hypoxia exposure and multiple sclerosis [21, 128]. Another pharmacological option is solifenacin, an FDA-approved anti-muscarinic drug primarily used for overactive bladder. Solifenacin has been proposed as a pro-myelinating drug, as it has been shown to enhance the differentiation of OPCs and promote myelin synthesis [129]. Notably, co-administration of solifenacin with donepezil, a medication used to treat AD-like memory loss and confusion, improved the efficacy of donepezil in cognitive performances [130]. Given these promising effects, the therapeutic potential of these compounds for AD warrants further studies.

Cell-based therapies also show promising prospects for myelin repair. Previous studies have demonstrated the efficacy of stem cell transplantation in promoting remyelination in both *in vitro* and *in vivo* models [131-133]. The transplantation of neural stem cells has been shown to lead to the effective replacement of the defective myelin while also promoting the remyelination of axons [134]. Experimental evidence also shows that when transplanted into mice with dysmyelination, human neural stem cells can differentiate into oligodendrocytes and boost functional myelination [135, 136]. In a clinical trial, the engraftment of neural stem cells resulted in increased brain myelin content in patients with Pelizaeus-Merzbacher disease, a disorder characterized by insufficient myelination in the brain [137]. Similarly, transplantation of OPCs has shown promise in promoting oligodendrogenesis and remyelination in experimental brain injuries [138-140]. Hence, promoting myelin repair by cell-based therapies may also present a valuable intervention for AD.

It should be noted that while pharmacological and genetic approaches effectively boosted myelin renewal in experimental animals, there are currently no interventions available to prevent myelin degeneration. Given the limited effectiveness of current AD therapy, it may be more important to develop preventive approaches that can maintain the integrity of myelin. Such interventions could potentially slow or even halt the progression of AD. In this regard, physical exercise represents a non-pharmacological approach that effectively prevents the loss of myelin during the progression of AD [141-143]. Compared to age-matched controls, animals that underwent long-term running training showed larger myelinated fiber volume and thicker myelin sheathes in the brain white matter [142, 143]. Therefore, elucidating the etiology of myelin degeneration in AD, as well as the molecular mechanisms underlying the positive effects of exercise on retaining myelin integrity, may provide novel insights into the development of myelin protection and repair strategies in AD.

Taken together, recent works in experimental animals have highlighted the potential of pro-myelinating approaches for AD-related cognitive deficits. While these results are encouraging, further clinical studies are required to reproduce these results in a clinical setting. Additionally, the development of both preventive and curative strategies for myelin pathology will advance future research and direction in this field.

## Conclusions

Well-functioning myelin is essential for the propagation of action potential, memory consolidation, and cognition. Myelin degeneration has been increasingly proposed as a major contributor to neurodegenerative diseases, which can be an early pathologic event during the progression of AD. Recent findings provide new insights into the role of myelin pathology in AD-associated memory loss. Mechanistically, DNA damage, Aβ toxicity, cholesterol dysregulation, iron/copper dysregulation, inflammation, and oxidative stress may be implicated in myelin pathology in AD. Furthermore, there is an overlap between cellular events driving myelin degradation and classic AD pathology. These cellular events may interact and trigger or exacerbate the progression of AD. More importantly, several pro-myelinating approaches have demonstrated promising results in animal models of AD. These findings indicate that myelin repair represents a therapeutic opportunity for AD, informing a new direction and therapy basis for therapeutic interventions. Nevertheless, considering the polyetiological origin of AD, single-target therapeutic approaches may have limited effects in mitigating or halting disease progression. Therefore, in addition to assessing the clinical safety and efficacy of pro-myelinating approaches, further research is needed to evaluate the efficacy of multi-target therapy in the management of AD.
